# Peripheral Inflammation and Insulin Resistance: Their Impact on Blood–Brain Barrier Integrity and Glia Activation in Alzheimer’s Disease

**DOI:** 10.3390/ijms26094209

**Published:** 2025-04-29

**Authors:** Teresa Ponce-Lopez

**Affiliations:** Centro de Investigación en Ciencias de la Salud (CICSA), Facultad de Ciencias de la Salud, Universidad Anáhuac México Campus Norte, Huixquilucan 52786, Mexico; teresa.ponce@anahuac.mx

**Keywords:** blood–brain barrier, Alzheimer’s disease, type 2 diabetes, chronic systemic inflammation, insulin resistance, proinflammatory cytokines, insulin signaling, microglia, neuroinflammation, amyloid beta, tau hyperphosphorylation, brain insulin resistance

## Abstract

Alzheimer’s disease (AD) is a progressive neurodegenerative disorder characterized by cognitive decline, memory impairment, and synaptic dysfunction. The accumulation of amyloid beta (Aβ) plaques and hyperphosphorylated tau protein leads to neuronal dysfunction, neuroinflammation, and glial cell activation. Emerging evidence suggests that peripheral insulin resistance and chronic inflammation, often associated with type 2 diabetes (T2D) and obesity, promote increased proinflammatory cytokines, oxidative stress, and immune cell infiltration. These conditions further damage the blood–brain barrier (BBB) integrity and promote neurotoxicity and chronic glial cell activation. This induces neuroinflammation and impaired neuronal insulin signaling, reducing glucose metabolism and exacerbating Aβ accumulation and tau hyperphosphorylation. Indeed, epidemiological studies have linked T2D and obesity with an increased risk of developing AD, reinforcing the connection between metabolic disorders and neurodegeneration. This review explores the relationships between peripheral insulin resistance, inflammation, and BBB dysfunction, highlighting their role in glial activation and the exacerbation of AD pathology.

## 1. Introduction

Alzheimer’s disease (AD) is a progressive neurodegenerative disorder characterized by cognitive decline, memory loss, and synaptic dysfunction, primarily affecting the elderly population [1]. It accounts for 60–70% of dementia cases in older adults [2]. The pathological hallmarks of AD include extracellular amyloid beta (Aβ) plaques and intracellular neurofibrillary tangles (NFTs), which are composed of hyperphosphorylated tau proteins, leading to neuronal dysfunction and synaptic loss [3,4,5].

Aβ is generated from amyloid-β precursor protein (APP) through sequential cleavage by β-secretase (BACE-1) and γ-secretase, forming highly fibrillogenic β-sheet structures [6,7]. In addition to neuronal production, Aβ can enter the brain via receptor-mediated transcytosis across the blood–brain barrier (BBB) [8]. Aβ plaques are initially deposited in the neocortex, particularly in the medial prefrontal and parietal regions [9]. The medial prefrontal is associated with Brodmann areas 9 and 10 and the anterior cingulate cortex, which is involved in decision-making, social behavior, and emotional regulation. The medial parietal regions involve the precuneus, posterior cingulate cortex, and Brodmann areas 7 and 31, which are implicated in processing spatial information, sensory integration, and aspects of memory [9]. These deposits can occur 10–20 years before clinical symptoms emerge [10]. Likewise for the association areas of the limbic cortex [11]; in these areas, Aβ causes mitochondrial and synaptic damage by altering cellular functions, including tau protein hyperphosphorylation [12]. Physiological levels of Aβ modulate neurogenesis, synaptic plasticity, and APP homeostasis. However, excessive Aβ production, aggregation, and deposition can impair biologically essential pathways that lead to neuronal cell death [13].

Tau protein stabilizes microtubules that help maintain shape and intracellular transport in neurons. Under pathological conditions, it undergoes specific alterations, the most common being phosphorylation at different residues, so its functionality is closely related to its phosphorylation level [14]. NFTs are primarily located in the hippocampus and cortical areas of the brain, leading to synaptic function loss, mitochondrial damage, microglia activation, and ultimately, neuronal death [15], which affects hippocampal circuits, leading to poor short- and long-term memory consolidation [16]. Glycogen synthase kinase-3β (GSK-3β) is one of the key kinases in regulating tau phosphorylation at Ser202/396 and Thr205 residues [17]. The overexpression of GSK-3β increases Aβ production and can suppress acetylcholine (ACh) synthesis [18] by causing inactivation of acetylcholine transferase (AChT) in the striatum, the basal nucleus of Meynert, and the frontal cortex [18]. Its activity is elevated in the brains of animal models and AD patients, contributing to pathology, cognitive impairment, and glial cell proliferation [19].

Neuroinflammation has emerged as a core feature of AD, playing a crucial role in its onset and progression [20,21]. It is critical to AD initiation, progression, and development [21]. It is an inflammatory response in the central nervous system (CNS), marked by increased resident macrophages and activation of glia cells (astrocytes and microglia), which play a crucial role in recognizing and eliminating toxic components following brain injury [21,22,23]. Activated glial cells in the brain enhance the production of inflammatory cytokines, including interleukin-6 (IL-6), interleukin-1β (IL-1β), and tumor necrosis factor-alpha (TNF-α) [24]. Evidence from AD brains reveals elevated cytokine levels and gliosis in affected regions [25,26]. A meta-analysis reported elevated blood levels of IL-6, IL-1β, and TNF-α in AD patients [27].

Eventually, chronic neuroinflammation can increase the production of proinflammatory IL-6, IL-1β, TNF-α, and other inflammatory mediators; exacerbate neuronal damage; and disrupt insulin signaling pathways, leading to brain insulin resistance—a condition linked to sporadic AD and its progression [28,29,30]. Insulin in the CNS regulates glucose metabolism, neuronal survival, synaptic plasticity, and cognitive processes [31]. Brain insulin resistance results from impaired insulin signaling, mainly through the insulin receptor substrate 1 (IRS-1)/phosphatidylinositol 3-kinase (PI3K)/protein kinase B (Akt) pathway [32,33]. This impairment reduces glucose uptake in neurons and promotes Aβ plaque formation by reducing Aβ degradation and clearance. It mainly affects the downstream insulin signaling pathway PI3K/Akt, thereby leading to increased production of Aβ and hyperphosphorylated tau [33]. Besides, brain insulin resistance enhances neuroinflammatory responses by activating inflammatory signaling pathways such as nuclear factor kappa B (NF-kB) and stress-activated c-Jun N-terminal kinases (JNKs), creating a detrimental feedback loop that accelerates neurodegeneration [34].

Emerging evidence suggests that peripheral inflammation and insulin resistance could be critical contributors to disease onset and progression, impacting both BBB integrity and glial cell activation. They are the major hallmarks of metabolic disorders such as type 2 diabetes (T2D), obesity, and hyperlipidemia, with an increased risk of AD [35,36]. T2D is one of the most critical metabolic disorders, characterized by prolonged high blood glucose levels, often resulting from the dysfunction of insulin-secreting β cells [35] and/or insulin resistance in peripheral tissues, which indicates decreased sensitivity to insulin so that it can carry out its mechanisms of action [37,38].

Risk factors for T2D include obesity (particularly visceral fat deposition), physical inactivity, being male, advanced age, certain ethnicities, sleep deprivation, urbanization, and low socioeconomic status [39]. Obesity is a significant risk factor for T2D and is strongly associated with insulin resistance, chronic low-grade inflammation, and metabolic disturbances [39]. Additionally, it is linked to metabolic syndrome, hypertension, and dyslipidemia, all of which correlate with increased levels of inflammatory biomarkers [40]. These conditions often induce chronic subacute inflammation, activating key inflammatory pathways such as JNK [41]. This inflammatory response is further amplified by the release of proinflammatory cytokines (IL-6, IL-1β, TNF-α) and adipokines, contributing to obesity-related metabolic and cardiovascular disorders [40]. Elevated levels of circulating proinflammatory molecules can compromise BBB integrity by disrupting tight junction proteins, facilitating neurotoxicity, and allowing inflammatory mediators such as reactive oxygen species (ROS), matrix metalloproteinases (MMPs), and nitric oxide (NO) to infiltrate the CNS [42,43,44]. This infiltration can activate microglia and astrocytes, leading to sustained neuroinflammation [45,46]. As a result, this process intensifies neuroinflammation, leading to sustained activation of glial cells and further exacerbating neuronal damage and cognitive decline.

The above is strongly supported by epidemiological studies that suggest T2D is a significant risk factor for developing cognitive dysfunction and AD [47,48,49]. For instance, insulin resistance is commonly associated with metabolic disorders associated with impaired cognitive function [28]. T2D is consistently linked to a higher risk of various cognitive issues, including dementia (AD and vascular dementia); mild cognitive impairment (MCI), a precursor to dementia; and overall cognitive decline, which is a crucial characteristic of dementia [50]. Since the Rotterdam study, a clinical report investigating the relationship between T2D and sporadic AD demonstrated that those patients diagnosed with diabetes had an increased and almost doubled risk of AD [51]. A meta-analysis including around 1.7 million people from 17 studies informed a relative risk (RR) of 1.53 (95% CI 1.42–1.63) for an occurrence diagnosis of AD in people with diabetes compared with people without diabetes [52]. Experimental models of diabetes and obesity exhibit AD-like neuropathological changes, including Aβ accumulation and tau abnormalities [53,54,55]. Thus, mechanisms analogous to those explaining peripheral insulin resistance in T2D may contribute to impaired brain insulin signaling and neuronal dysfunction in AD [48]. Considering the complex association between peripheral inflammation, insulin resistance, BBB dysfunction, and glial cell activation, this review seeks to provide a comprehensive analysis of their interconnected roles in AD pathology.

## 2. Peripheral Insulin Resistance

Insulin plays a pivotal role in glucose metabolism and energy homeostasis in peripheral tissues by facilitating glucose uptake via glucose transporter type 4 (GLUT4) translocation, promoting glycogen synthesis in the liver and muscles, and stimulating lipid and protein synthesis while inhibiting lipolysis and gluconeogenesis. By maintaining blood sugar levels and interacting with hormones like glucagon, insulin ensures a balanced regulation of energy storage and utilization across the body [56].

Insulin resistance is the main hallmark of obesity and T2D [57]. Insulin resistance is when a cell or tissue cannot correctly respond to regular amounts of insulin. Thus, insulin levels are normal but fail to activate the signal for glucose uptake, forcing the body to overstimulate pancreatic β cells, causing hyperinsulinemia to maintain optimal blood glucose levels [58,59]. Eventually, the pancreatic β-cell pool is overwhelmed by the metabolic demands to maintain euglycemia, establishing sustained hyperglycemia that eventually progresses to T2D, a condition associated with increased body fat [60]. The tissues that develop insulin resistance are adipose tissue, muscle, and liver, and it is believed to occur 10 to 15 years before the onset of T2D [61]. Hyperinsulinemia is likely a late consequence of insulin resistance—having abnormally high insulin levels in the bloodstream. It is usually a compensatory response to hyperglycemia or insulin resistance that reduces the number of insulin receptors and their function [57]. Several factors facilitate insulin resistance, such as age, increased adiposity, decreased muscle mass, and a sedentary lifestyle [62]. Therefore, insulin resistance is among the first contributing metabolic abnormalities that leads to other metabolic developments, like hyperglycemia, dyslipidemia, visceral adiposity [63], high inflammatory markers, and endothelial dysfunction [64].

Some research indicates that high levels of free fatty acids (FFAs) during fasting and after meals may elevate the risk of developing T2D and obesity [65]. In insulin-resistant visceral adipose tissue, insulin fails to suppress lipolysis by increasing circulating FFAs, affecting hepatic and muscle metabolism and aggravating insulin resistance [66]. Also, these temporarily raise β-cell mass and insulin output to counteract the reduced sensitivity to insulin. However, a sustained elevation in plasma FFA levels leads to imbalances in the regulation of lipid metabolism. It negatively affects β-cell function and survival due to lipotoxicity, ultimately contributing to the onset of T2D [67]. FFAs impact the insulin signaling pathway by elevating diacylglycerol (DAG) levels, ROS, and protein kinase C (PKC). These changes subsequently enhance serine phosphorylation and reduce the tyrosine phosphorylation of the IRS-1. As a result, PI3K/Akt/AS160 activity is suppressed, thus reducing the translocation of GLUT4 [68,69]. This disruption ultimately upsets the delicate equilibrium between the function of β cells and peripheral insulin resistance, culminating in the clinical onset of T2D [68].

### 2.1. Molecular Mechanisms of Insulin Resistance

#### 2.1.1. Insulin Signaling

**Figure 1:**
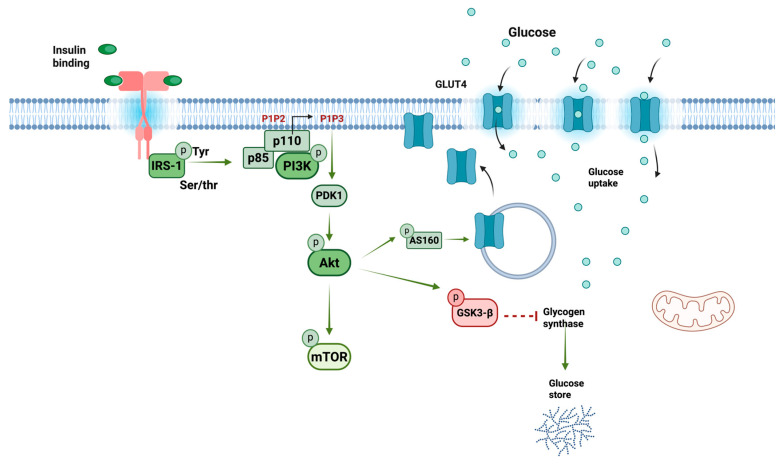
Insulin signaling pathway regulating glucose uptake and storage. Insulin binding to its receptor initiates a cascade of phosphorylation events, starting with the IRS-1 on Tyr residues, which activates PI3K through its regulatory (p85) and catalytic (p110) subunits. PI3K facilitates the conversion of PIP2 to PIP3, recruiting PDK1 and leading to the activation of Akt. Akt phosphorylates AS160, promoting the translocation of GLUT4 vesicles to the plasma membrane for glucose uptake. Simultaneously, Akt inhibits GSK3-β, enhancing glycogen synthase activity and glycogen storage. This process supports cellular energy homeostasis by regulating glucose entry and metabolism. Solid arrows: indicates activation or conversion step in the signaling pathway. Dotted lines: indicates inhibitory effect. IRS-1: insulin receptor substrate-1; Tyr: tyrosine residues; PI3K: phosphoinositide 3-kinase; PIP2: phosphatidylinositol 4,5-bisphosphate; PIP3: phosphatidylinositol 3,4,5-trisphosphate; PDK1: phosphoinositide-dependent kinase-1. GLUT4: glucose transporter 4; GSK3-β: glycogen synthase kinase-3 beta.

Insulin signaling is crucial in regulating glucose balance and managing its metabolism in the liver, muscle, and fat tissues. However, inflammation and proinflammatory cytokines can impact insulin signaling, altering glucose absorption [70]. The insulin receptor (IR) is a transmembrane glycosylated, disulfide-linked α2β2 tetramer that belongs to a subfamily of receptor tyrosine kinases. IR is present on the surface of nearly all tissues, with significant influence on insulin-sensitive organs like the liver, muscle, and adipose tissues [71]. The receptor becomes active when insulin binds to the α subunit, unlocking the tyrosine kinase activity in the β subunit [Figure 1]. Its structure is divided, having an extracellular segment for insulin binding and an intracellular part linked with its tyrosine kinase activity [72]. Insulin binding to the α subunit of the extracellular section leads to its dimerization into α2β2, activating the self-phosphorylation of the beta subunit at positions pTyr1158, pTyr1162, and pTyr1163 [73]. The IR tyrosine kinase activation prompts the recruitment and phosphorylation of various substrates, such as insulin receptor substrates 1-4 (IRS1-4). IRS-1/2 are the most common and vary significantly in abundance and tissue-specific roles. Despite the high structural similarity, both differ in activation sites and cellular localization [73]. IRS-1 plays a more critical role than IRS-2 in skeletal muscle, an essential site for whole-body metabolism [74]. The expression of IRS-1 is significantly reduced in individuals who are obese or insulin-resistant [75]. These proteins provide specific binding sites to attract other proteins to different downstream pathways [76]. The phosphorylation of the tyrosine residue of IRS-1 (IRS-1pTyr) activates the p85 regulatory subunit of PI3K (a class 1 enzyme), which, in turn, induces a conformational change of this protein that leads to the binding of the catalytic subunit p110 that activates PI3K; once activated, this protein phosphorylates phosphatidylinositol (4,5)-biphosphate (PIP2) at the cell membrane and induces the production of phosphatidylinositol (3,4,5)-trisphosphate (PIP3) [76]. PIP3 subsequently allows the activation of protein kinases that contain the pleckstrin homology (PH) domain. These kinases activate phosphoinositide-dependent kinase 1 (PDK1) and Akt. These proteins are then transported to the cell membrane, where Akt is phosphorylated at threonine 308 (pThr308) by PDK1 activity [77].

Once activated, Akt moves to the plasma membrane and phosphorylates the Akt substrate (AS160) [78], leading to the translocation of GLUT from internal storage vesicles to the plasma membrane [79]. Then, Akt phosphorylates and inactivates GSK3, which leads to the activation of glycogen synthase and glycogen accumulation in the liver [80]. Furthermore, Akt phosphorylates Proline Rich Akt Substrate, 40 kDa (PRAS40), an inhibitor of the mammalian target of rapamycin (mTORC1), thus lifting the inhibition. This allows the mTORC1 complex to phosphorylate and inhibit 4E-binding protein 1 (4E-BP1), activate ribosomal protein S6 kinases 1,2 (S6K1, S6K2) and sterol regulatory element binding protein (SREBP1), and regulate a network of genes that control metabolism, protein synthesis, and cell growth [81]. Another effector that Akt could phosphorylate is the O forkhead box-containing transcription factor (FoxO). Akt eliminates FoxO from the nucleus, inhibiting its transcriptional activity. Then, the Akt/Foxo1 axis facilitates the insulin-mediated regulation of hepatic glucose production [82].

#### 2.1.2. Insulin Signaling Disruption

Insulin resistance happens mainly at the level of insulin-sensitive tissues, such as the liver, muscle, and fat, and can be caused by multiple mechanisms. It could result from alterations of insulin signaling due to either internalization of IR or post-translational modifications of downstream protein effectors [34,72].

Upon receptor internalization, IR regulates the response to insulin at the cell surface through processes such as endocytosis and receptor recycling. This negative regulation of IR is a way to control the availability of these receptors at the cell surface [83]. Specific genetic mutations in IR can even result in severe insulin resistance. For instance, in T2D patients, the rate of IR internalized into cells is impaired compared to type 1 diabetes patients with normal insulin sensitivity [84]. Failure in IR internalization can result in exacerbating insulin resistance and hyperinsulinemia. It has been observed in patients with a specific mutation in IR where arginine is replaced by cysteine at position 252 [85]. However, in rare instances, mutations in the IR gene can cause significant insulin resistance, where affected individuals might need over 100 times the insulin amount typically required by diabetic patients [72].

Changes in the post-translational modifications of downstream protein effectors by the activation of various serine/threonine kinases is a critical process in tissues related to insulin resistance, particularly in T2D, where hyperinsulinemia can suppress the expression of IRS-1/2, and factors like degradation and reduced synthesis can lead to lower protein levels of these mediators. Moreover, the insulin-degrading enzyme (IDE) plays a vital role in managing external insulin levels, thereby influencing IR signaling [57,86]. In an animal model of hyperinsulinemia secondary to a high-fat diet (HFD), the insulin gene (Ins2 gene) deletion protected rats from obesity secondary to diet in the absence of hyperinsulinemia and insulin resistance [87]. Previously, evidence revealed that IR function disruptions can lead to insulin resistance [88].

**Figure 2:**
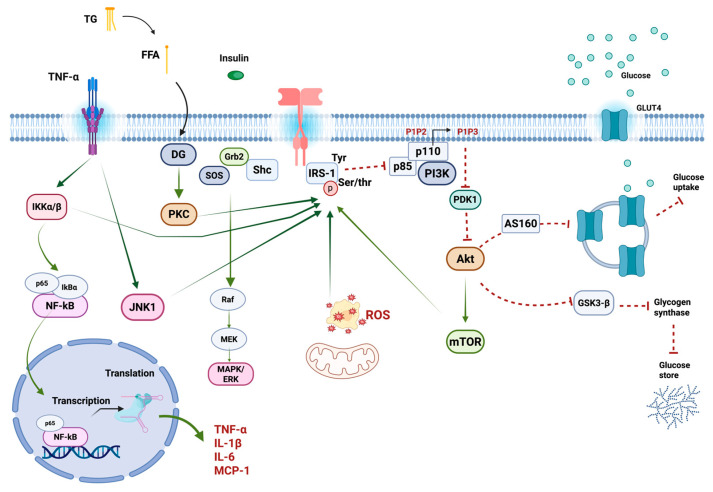
Interaction between insulin signaling, inflammation, and oxidative stress in cellular metabolism. Insulin binding to its receptor activates IRS-1, PI3K, and Akt pathways, promoting GLUT4 translocation for glucose uptake and glycogen storage. Simultaneously, TNF-α signaling through its receptor triggers proinflammatory cascades via NF-κB and JNK1, which inhibit IRS-1 activity through serine/threonine phosphorylation. Elevated levels of FFAs stimulate the DG-mediated activation of PKC, further disrupting insulin signaling. Additionally, TNF-α enhances ROS production and NF-κB activation, leading to increased transcription of inflammatory cytokines (TNF-α, IL-1β, IL-6, MCP-1). This crosstalk between insulin resistance, inflammation, and oxidative stress contributes to metabolic dysfunction and impaired glucose homeostasis. Solid arrows: indicates activation or conversion step in the signaling pathway. Dotted lines: indicates inhibitory effect. GLUT4: glucose transporter 4; IRS-1: insulin receptor substrate-1; PI3K: phosphoinositide 3-kinase; TNF-α: tumor necrosis factor alpha; NF-κB: nuclear factor kappa B; JNK1: c-Jun N-terminal kinase 1; FFAs: free fatty acids; DG: diacylglycerol; PKC: protein kinase C; ROS: reactive oxygen species; IL-1 β: interleukin-1β; IL-6: interleukin-6; MCP-1.

Identifying the specific protein kinases involved in obesity-induced chronic inflammation could help understand insulin resistance. IRS features an N-terminal PH domain surrounded by the phosphorylation of the tyrosine binding (PTB) domain, facilitating its interaction with the insulin receptor. The tail of IR houses unique tyrosine and serine phosphorylation sites, serving as the docking point for SH2 homology signal transducers. [71]. The serine/threonine phosphorylation of IRS-1 (IRS-1pSer/Thr) causes the dissociation of the IR and IRS-1 complex, which is related to the inhibition of IRS-1pTyr [72]. Hence, the balance of IRS-1pTyr and IRS pSer/Thr protein determines the magnitude of insulin actions and plays a fundamental role in the progression of insulin resistance [42] (Figure 2).

In animal models with insulin resistance, there is increased activation of the mTOR pathway and enhanced IRS-1Ser307/636/639. This elevated Ser phosphorylation of IRS-1 is absent in S6K1-deficient mice [89]. Therefore, the activation of the mTOR pathway, which negatively affects IRS-mediated signaling, is acknowledged as a feedback mechanism potentially contributing to the development of insulin resistance in vivo [90]. Afterward, IRS-1pSer/Thr increases the inflammatory response to produce proinflammatory cytokines, FFAs, hyperglycemia, mitochondrial dysfunction, and endoplasmic reticulum stress. This response occurs through activating numerous kinases, primarily JNK1, IκB kinase (IKK), PKC, mTORC1/S6K, and MAPK [72,91]. These stress kinases interfere with IRS-1 function, promoting insulin resistance by increasing inflammation-related gene expression and the activation of nuclear factor kappa B (NF-κB) and intensifying insulin resistance [91].

### 2.2. Impact of Impaired Mitochondria

The mitochondria within cells are a significant site of ROS and reactive nitrogen species (RNS) production and, therefore, play a central role in oxidative stress. ROS and RNS are created as part of normal cell physiology during cellular respiration within the mitochondria. ROS is a term used to describe a group of reactive molecules and free radicals derived from oxygen. These molecules are highly reactive due to the presence of unpaired electrons. For instance, ROS include superoxide (O2•−) and hydroxyl (OH), which are radicals, and hydrogen peroxide (H2O2), which is not a radical. These can also be further classified based on whether they are ions. O2 is an ion, while H2O2 is not [92]. Nitrogen-derived RNS include ions like peroxynitrite (ONOO−) and non-ions like nitric oxide (NO). NO is generated during the breakdown of arginine to citrulline by a family of NADPH-dependent enzymes called nitric oxide synthases (NOSs) [93].

These compounds can influence mitochondrial functions in various ways. These include stimulating mitochondrial biogenesis, damaging the mitochondrial DNA, instigating the oxidation of lipids, and altering the permeability of the mitochondria [92,94]. Mitochondria play a pivotal role in utilizing fats for energy production, and any decline in mitochondrial function is closely related to the accumulation of ectopic fat and the emergence of insulin resistance. Studies have shown that resting adenosine triphosphate (ATP) synthesis in skeletal muscle is notably reduced in insulin-resistant individuals compared to their insulin-sensitive counterparts, implying a substantial contribution of mitochondrial dysfunction to IR [95].

The intricate interaction between mitochondrial dysfunction and oxidative stress is a key factor associated with insulin resistance and T2D [96]. The combination of elevated fatty acids and hyperglycemia intensifies the production of mitochondrial ROS and further suppresses mitochondrial function [97]. Mitochondrial ROS diminish insulin responsiveness in adipocytes in mice [98]. They disrupt insulin-triggered GLUT4 translocation in cells by interfering with the insulin-induced redistribution of IRS-1 and PI3K [99]. Studies on obese, insulin-resistant humans and HFD rats have revealed an elevated emission of H2O2 from skeletal muscle mitochondria, suggesting a correlation between mitochondrial H2O2 production and the development of insulin resistance [100].

### 2.3. Role of Advanced Glycation End Products

In conditions of insulin resistance, hyperglycemia is a prevalent feature contributing to diabetic complications. It is also involved in a series of non-enzymatic chemical processes known as the Maillard reaction, which produces advanced glycation end products (AGEs) [101]. AGEs are heterogeneous compounds derived from irreversible non-enzymatic sugar reactions with the amino groups of protein, nucleotides, and lipids. This process usually occurs over years and is accelerated in patients with T2DM due to the increased formation of ROS [102]. AGEs alter the structure of proteins, plasma lipoproteins, cell membrane phospholipids, or DNA, potentially leading to the formation of aggregated proteins and thus contributing to the toxicity of AGE-modified polypeptides [103].

AGE production can be both endogenous and exogenous, with diet also playing a role in contributing to the risk of insulin resistance. The damaging effects of AGEs occur through their interaction with the AGE receptor (RAGE), a receptor expressed on the cell surface of many cell types [104]. Thus, RAGE plays a significant role in the development of pancreatic β-cell dysfunction and the progression of diabetes. Increased levels of circulating AGEs can elevate the expression of RAGE in the pancreas, which, when engaged with toxic protein aggregates, contributes to islet amyloidosis, a pathology characterized by the aggregation of the hormone islet amyloid polypeptide (IAPP) or amylin, a hormone produced by pancreatic β cells [105]. The interaction of RAGE and IAPP, or amylin, activates oxidative stress, inflammation, and apoptosis, all critical features of IAPP-induced β-cell cytotoxicity [106].

Moreover, the chronic activation of RAGE leads to the upregulation and activation of NF-κB, which elevates the expression of RAGE itself and proinflammatory cytokines. These cytokines initiate endoplasmic reticulum stress, activating the unfolded protein response to restore cellular homeostasis [106]. The subsequent inflammation, oxidative stress, and PKC activation inhibit IRS-1 by increasing IRS-1pSer/Thr and decreasing IRS-1pSer, impairing insulin signaling [107]. Specifically, the NF-κB p65/105 subunits bind to the promoter region of the solute carrier family 2-member 4 (Slc2a4) gene, thus downregulating the transcription of GLUT4, which plays a crucial role in glucose uptake [108].

## 3. Peripheral Inflammation

Peripheral inflammation is the most important factor that might cause or worsen insulin resistance and T2D. The pathogenesis of T2D likewise implicates inflammatory processes, where the low-grade systemic inflammation could produce insulin resistance in most insulin-sensitive tissues [109]. Inflammation is critical in the pathophysiology of T2D, obesity, and related metabolic disturbances. In obese patients, augmented inflammatory markers have been observed, for example, C-reactive protein (CRP) and proinflammatory cytokines such as TNF-α, IL-6, and IL-1β [108]. Higher concentrations of inflammatory mediators are related to insulin resistance in people at risk of T2D.

A randomized clinical trial demonstrated that elevated CRP and IL-6 levels were associated with the development of T2D, suggesting their importance as inflammatory markers in diabetogenesis [110]. Obesity has been positively associated with levels of inflammatory biomarkers, which predict insulin resistance and the incidence of T2D [41,111,112]. Excessive caloric intake and insufficient physical activity accumulate fat in the subcutaneous tissue and, over time, in the liver, pancreas, muscles, perivascular tissues, and pericardium [113]. Consequently, insulin resistance increases tissue and β-cell pancreatic dysfunction [114]. Fat accumulation in the liver (steatosis) preceding T2D is associated with obesity and reduced hepatic insulin sensitivity, leading to fasting hyperglycemia [113,115]. Low chronic inflammation is a common cause due to the activation of at least two main inflammatory pathways: stress-activated c-Jun N-terminal kinase (JNK) and NF-kB [41,116].

Data from studies showed that brown adipose tissue regulates glucose and energy homeostasis and is associated with insulin resistance and hyperglycemia [117,118]. In contrast, visceral white adipose tissue produces cytokines and other substances involved in inflammatory pathways such as TNF-α, IL-6, IL-1β, interleukin-10 (IL-10), leptin, adiponectin, monocyte chemoattractant protein, chemokines, and serum amyloid protein, known collectively as adipokines [119,120,121].

### 3.1. Macrophage’s Role

Additionally, macrophages, immune cells (B and T cells), and adipose tissue infiltration trigger chronic low-grade local and systemic inflammation by producing more cytokines and chemokines [122]. There are two distinct phenotypes of macrophages: M1 and M2. M1 macrophages produce proinflammatory cytokines like TNF-α, IL-6, and monocyte chemoattractant protein (MCP-1), which reduce insulin signaling in different tissues, thus contributing to insulin resistance and the onset of T2D. Meanwhile, M2 macrophages, found in lean fat, are stimulated by and secrete IL-10, a potent anti-inflammatory cytokine involved in tissue repair and remodeling. The balance between M1 and M2 macrophages determines the overall inflammatory effect and, therefore, the level of insulin sensitivity [123]. When adipose tissue recruits macrophages due to the overexpression of MCP1, which is a chemokine ligand known as CCL2, liver insulin resistance is observed with increased TNF-α expression in adipose tissue. This happens without any changes in body weight or fatness. Moreover, removing MCP1 has shown protection against insulin resistance induced by a high-fat diet [124]. Similarly, the inactivation of C-C chemokine receptor type 2 (CCR2), a chemokine receptor on the surface of specific immune cells, and treatment with INCB3344 (a CCR2 antagonist) increased insulin sensitivity. It decreased the recruitment of macrophages from adipose tissue [125].

### 3.2. TNF-α as a Mediator of Inflammation and Insulin Resistance

Among the different proinflammatory cytokines, TNF-α is one of the most significant mediators involved in insulin resistance development and the pathogenesis of T2DM [126,127,128]. TNF-α overexpression in the adipose tissue of obese individuals [129] and obese mice [130] causes peripheral insulin resistance. Activated macrophages produce TNF-α and regulate immune cells. TNF-α overexpression in adipose tissue decreases peripheral glucose uptake in response to insulin, leading to insulin resistance [131]. Moreover, TNF-α has been demonstrated to increase glucose and triglyceride production in the liver, meanwhile leading to insulin resistance by a reduction in peripheral glucose uptake in response to insulin [131].

TNF-α promotes inflammation and insulin resistance by influencing the MAPK and NF-κB signaling pathways and IRS-1 phosphorylation. TNF-α activates the adipocyte signaling pathway, which is essential for inflammation and insulin resistance [132]. In these cells, TNF-α stimulates the activity of MAPKs like p38 and JNK [133], as well as NF-κB [134]. NF-κB is triggered by various stimuli, including cytokines and oxidative stress, and plays a crucial role in numerous processes, such as cell proliferation, inflammation, and apoptosis [135]. The intricate NF-κB signaling pathway comprises five distinct transcription factors: p65, p50, p52, c-Rel, and RelB. These factors possess a homology domain at their N-terminus, the Rel homology domain (RHD), facilitating interaction with DNA and dimerization with other RHD-containing factors. The primary regulation of NF-κB comes from inhibitory proteins known as inhibitor kappa B (IκB), which use this domain to keep the protein in the cytoplasm [136]. The initiation of the IκB kinase α/β (IKKα/β) by TNF-α leads to the phosphorylation of IκBα, causing it to dissociate from NF-κB, which is activated and translocated into the cell nucleus [136]. These events initiate the transcription of various proinflammatory molecules, such as IL-6, IL-1β, and MCP-1 [137]. Additionally, JNK1 and IKKα/β are activated, leading to the Ser307 phosphorylation of IRS-1 [138,139]. This disrupts normal insulin signaling and contributes to insulin resistance [140] (Figure 2).

### 3.3. Peripheral Inflammation and Its Contribution to Neuroinflammation in AD

Peripheral inflammation has emerged as a critical factor contributing to the onset and progression of neuroinflammation and AD. Chronic systemic conditions such as obesity, T2D, metabolic syndrome, aging, and infections are well-established sources of sustained peripheral inflammation. These conditions promote the overproduction of key inflammatory mediators like TNF-α, IL-6, and IL-1β, primarily by activated immune cells such as macrophages and monocytes. Once released into circulation, these cytokines can act on the BBB, a highly selective structure that regulates brain homeostasis [141,142].

Proinflammatory signals disrupt TJ proteins, enhancing endothelial cell activation [30]. Additionally, oxidative stress triggered by immune and endothelial cells disrupts the BBB structure by damaging essential lipids and proteins [143], increasing BBB permeability partly through activating the NF-κB signaling pathway [45].

Peripheral inflammation not only increases BBB permeability but also impairs its transport functions and contributes to cognitive decline and neurodegeneration [45]. Disrupted amino acid and peptide transport also alters neurotransmitter balance, aggravating neuronal injury [144].

**Figure 3:**
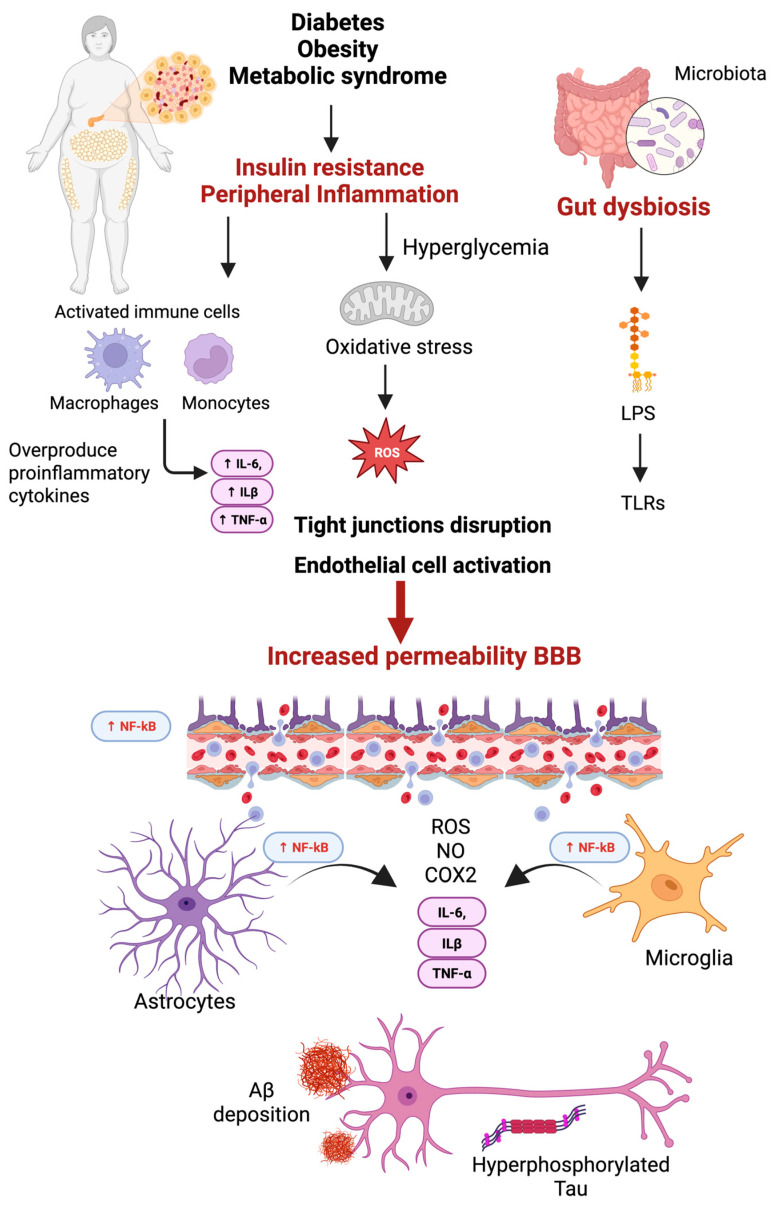
Peripheral inflammation-induced BBB disruption and neuroinflammation.

As the integrity of the BBB declines, circulating cytokines and other inflammatory mediators gain access to the CNS, where they contribute to glial cell activation, particularly of microglia and astrocytes. Activated glial cells release additional proinflammatory molecules, including ROS, NO, and cyclooxygenase-2 (COX-2), amplifying neuroinflammatory responses [30]. This inflammatory environment promotes synaptic dysfunction and facilitates the accumulation of Aβ plaques and the hyperphosphorylation of tau protein—two key neuropathological hallmarks of AD. Ultimately, these events culminate in progressive cognitive decline and neuronal loss [42] (Figure 3). This complex interrelationship is described in detail in the following sections.

## 4. BBB Disruption, Glia Activation, and Neuroinflammation

The brain’s neural environment must be kept within a strict homeostatic range to ensure normal functioning, maintaining a balance between the brain and peripheral circulation [145]. It demands stringent control over the transport of cells, molecules, and ions between the blood and the brain. The CNS forms a unique physiological and anatomical barrier to enforce this tight regulation. The BBB is an essential component of the cerebrovascular system. It is a critical function of brain blood microvessels to maintain CNS homeostasis [146,147].

### 4.1. BBB Structure, Function, and Regulation

**Figure 4:**
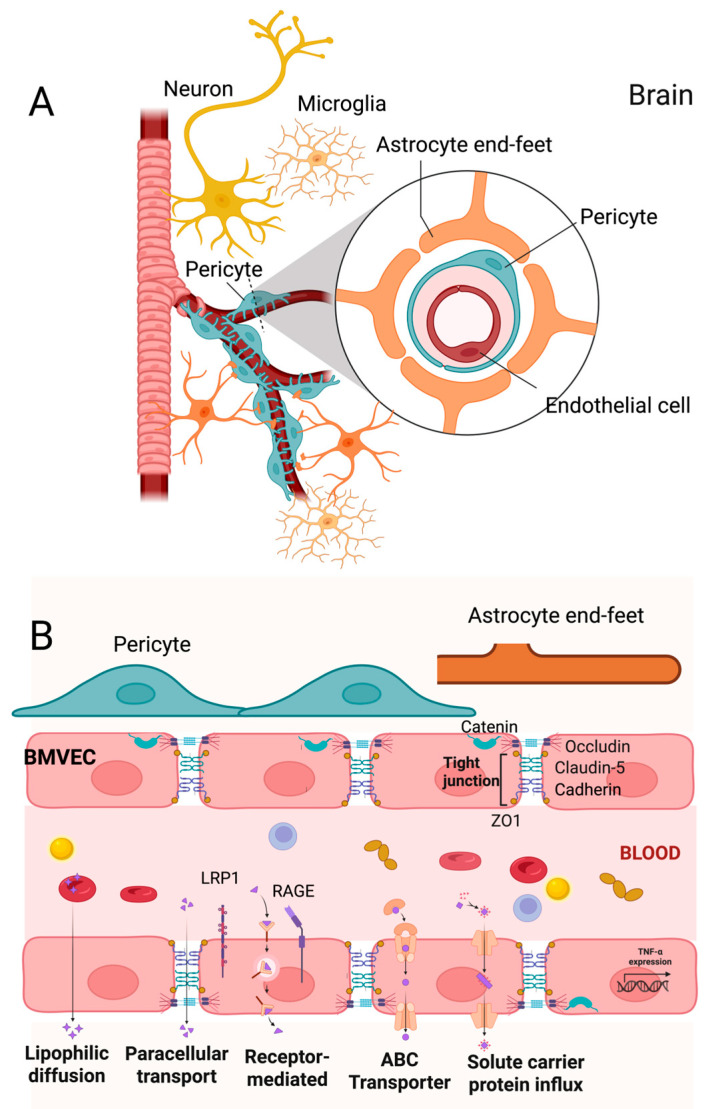
Structure and function of the blood–brain barrier. (**A**) The cellular composition of the BBB, highlighting its interactions with neurons, astrocytes, microglia, and pericytes. The inset shows a detailed cross-section of the neurovascular unit, where astrocyte end-feet and pericytes envelop the endothelial cells of the brain microvasculature. (**B**) Mechanisms of molecular transport across the BBB. BMVECs form a tightly regulated barrier reinforced by tight junction proteins (occludin, claudin-5, cadherin, ZO-1) and adherent junctions (catenin). Transport mechanisms include passive lipophilic diffusion, paracellular transport, receptor-mediated transcytosis, ABC transporters, and solute carrier protein influx. The expression of proinflammatory cytokines, such as TNF-α, can influence BBB permeability. Astrocyte end-feet and pericytes further modulate BBB function and integrity. BBB: blood–brain barrier; BMVECs: brain microvascular endothelial cells; ZO-1: zonula occludens protein 1; ABC: ATP-binding cassette; SC: solute carrier protein; TNF-α: tumor necrosis factor alpha.

The BBB, structurally defined as specialized endothelial cells lining the intraluminal side of brain capillaries, acts as a tightly regulated interface separating peripheral circulation from the CNS. Notably, each neuron is estimated to have a capillary [44]. Neurons require a steady supply of oxygen and nutrients, maintaining a precise balance in the brain since they are susceptible to the presence and concentration of various compounds [148]. Its interaction with astrocytes and microglia is essential for maintaining extracellular ionic balance, facilitating synaptic transmission, and regulating cerebral blood flow [149]. These neurons extend to all cells constituting the BBB [148]. The structure of the BBB is meticulously constructed to form a physical barrier through tight junctions between specialized endothelial cells in the blood vessels, forming what is known as the neurovascular unit [149]. Pericytes subsequently envelop the outer wall of the endothelium, the astrocytes’ end-feet, and microglia [150,151,152,153] (Figure 4A).

### 4.2. Endothelial Cells

Endothelial cells, particularly brain microvascular endothelial cells (BMVECs), bridge blood and the brain and help facilitate communication between the CNS and the peripheral nervous system by modulating the migration of circulating immune cells into the brain. Endothelial junctions between them are essential for preserving tissue integrity and maintaining vascular permeability (Figure 4B). These establish a polarized monolayer, distinguishing the luminal (apical) and abluminal (basolateral) compartments. These distinct regions separate the brain parenchyma from the peripheral system [154]. The endothelial cells of the BBB are complex structures, with tight junctions (TJs) and adherent junctions (AJs) playing crucial roles in their integrity. TJs comprise a transmembrane network of proteins, including occludin, claudins, and various zonula occludens (ZO) proteins (ZO-1, ZO-2, ZO-3), which create a tight seal between cells. At the same time, AJ comprises cadherin, catenins, vinculin, and actin [154,155,156]. TJs function as a border between the apical and basolateral plasma membrane domains and seal the paracellular space to control protein diffusion and cellular trafficking. This is required to ensure tissue integrity and regulate vascular permeability [157].

The luminal and abluminal compartments of endothelial cells express a wide range of transporters and receptors that are involved in the selective uptake of substances from the blood to the brain and form barriers, facilitate nutrient transport, support receptor-mediated signaling, control leucocyte traffic, and regulate osmotic pressure [154,158,159]. The functionality of the BBB is significantly influenced by the presence of various influx and efflux transporters expressed on endothelial cells, particularly those belonging to the solute carrier (SLC) superfamilies [160,161,162], ATP-binding cassette (ABC) transporters [163,164,165], and the major facilitator superfamily domain-containing protein 2a (MFSD2a), as specified in Table 1 [155], as well as the receptor low-density lipoprotein receptor-related protein 1 (LRP1) and RAGE, which mediate the transport of Aβ [166,167] and AGEs, [168,169] as provided in Table 2 (Figure 4B).

### 4.3. Pericytes

Pericytes, contractile cells found around capillary walls, are believed to regulate multiple functions of endothelial cells, such as their proliferation, survival, migration, differentiation, and vascular branching [170]. BBB restricts the entry of toxins, immune cells, and pathogens into the brain; it is vital for good neural function [171]. Additionally, endothelial cells are crucial for adult brain angiogenesis by secreting factors such as vascular endothelial growth factor (VEGF) and Notch ligands [172].

### 4.4. Astrocytes

Astrocytes, specialized glial cells mainly located in the white and grey matter of the brain, exist in fibrous and protoplasmic forms, respectively [173]. They are the most abundant cell type in the brain [174], known as the brain’s “nerve glue”, which covers 99% of the BBB’s endothelium and is essential for proper neuronal function. Within the neurovascular unit, astrocytes are strategically positioned between cerebral endothelial cells and neurons, with their end-feet ensheathing most capillary vessels [175,176]. They interact with the BMVECs within the neurovascular unit and participate in sonic hedgehog (Shh) signaling, reducing the expression of inflammatory mediators in brain endothelial cells [170]. These cells play a crucial role in maintaining the integrity of the BBB. Astrocyte-derived Shh signaling stimulates the expression of TJ proteins, including claudin-5 and occludins, in endothelial cells in vitro [177,178]. Additionally, the secretion of growth factors from astrocytic end-feet supports vascular integrity by upregulating nutrient transporters in endothelial cells [177]. These cells actively regulate brain functions such as BBB, neurogenesis, synaptogenesis, and neurotransmitter and fluid balance maintenance [179]. Moreover, astrocytes create specialized perivascular channels within the glymphatic system, acting as a clearance pathway for neurotoxic wastes like Aβ and tau proteins [173].

### 4.5. Microglia

Microglia are immune cells that protect the brain; they act immediately to manage changes in the brain. They are vital in pathological alterations in CNS diseases [180,181]. Microglia exist in two states or phenotypes. Under physiological conditions, the proinflammatory phenotype is called M1. It has a branched morphology that allows it to detect foreign bodies and neuronal lesions and is responsible for maintaining neuronal homeostasis, protection, and repair [182]. It protects the brain against pathogens and tumor cells by producing proinflammatory cytokines such as IL-1β, TNF-α, IL-6, IL-12, IL-23, and ROS. It is also associated with neuronal loss. The other phenotype, called M2, has a branched to amoeboid morphology like a macrophage and has anti-inflammatory properties. Under conditions such as infection, injury, ischemia, or inflammation, microglia become activated, proliferate, and change their morphology. [182,183]. Therefore, M2 promotes tissue remodeling/repair and angiogenesis by releasing anti-inflammatory cytokines such as IL-10, IL-4, IL-13, and TGF-β [184].

### 4.6. Blood–Brain Barrier Disruption

Peripheral inflammation is fundamentally a protective response; however, excessive and uncontrolled inflammation can lead to harmful consequences. The complex relationship between insulin resistance and inflammation in T2D and neurodegenerative disorders has garnered increased attention from the scientific community [185,186]. BBB disruption is linked to various comorbidities, including vascular conditions like atherosclerosis [187]. T2D, hypertension, obesity, and sleep disorders are also recognized as contributing factors to BBB disruption and AD progression [188,189].

Several studies suggest that chronic peripheral inflammation may cause an increase in proinflammatory cytokines in the CNS by crossing the BBB and eliciting an inflammatory response in microglia, contributing to neuroinflammation and cognitive impairment in patients with or without AD [190,191]. For instance, in obese patients, adipocytes produce proinflammatory cytokines (TNF-α, IL-1β, IL-6) and adipokines, and resident macrophages change the M1 phenotype (proinflammatory state) [192]. A meta-analysis revealed that peripheral inflammatory markers correlate with dementia risk [193]. The Framingham study reported that higher peripheral IL-1β and TNF-α might be markers of AD risk [194]. Circulating levels of these proinflammatory mediators and CRP are relevant biomarkers linking obesity and T2D with late-onset AD [195]. Specific genetic variants cause increases in proinflammatory cytokines (IL-1β, IL-6, and TNF-α) and are associated with an increased risk of developing AD [196]. Postmortem brains of T2D patients with AD showed elevated levels of IL-6 compared to control brains [184]. Elevated IL-6 levels are a relevant predictor of executive dysfunction in people with high BMI [197].

In vivo studies report that in animals in which obesity was induced with a high-fat diet, there was increased BBB permeability, hippocampal-dependent cognitive dysfunction in rats [198], and microglial activation in mice [199]. Additionally, in a transgenic mouse model of AD, the BBB is more permeable to peripheral inflammatory cytokines [200].

## 5. Mechanisms Linking AD to BBB Breakdown

The BBB depends on a balance between transcellular and paracellular transport pathways to maintain brain homeostasis. TJs are critical for regulating paracellular transport, and their dysfunction is implicated in neurodegenerative diseases like AD. TJ dysfunction can disrupt this balance, increasing BBB permeability and neuronal damage. Infectious and non-infectious pathological conditions, including diabetes, AD, Parkinson’s disease (PD), trauma, stroke, epilepsy, multiple sclerosis, and tumors, can disrupt the integrity of the BBB by affecting biochemical substance concentrations [171,201].

In T2D, insulin resistance and chronic hyperglycemia could contribute to endothelial dysfunction and inflammation, compromising BBB integrity and potentially facilitating neurotoxin entry, such as Aβ [202,203]. These molecules may trigger glial activation, brain inflammation, and neuronal insulin resistance [32]. Without direct inflammation, this can lead to brain dysfunction and cognitive decline [195]. Notably, inflammatory processes in the brain mirror those in peripheral tissues [32]. Similarly, obesity induces systemic inflammation and endothelial dysfunction, exacerbating BBB impairment [204]. These conditions frequently co-occur, compounding their effects on BBB dysfunction and pathology [205]. They promote TJ degradation, increasing the risk of BBB breakdown and AD progression. This establishes a vicious cycle: BBB dysfunction leads to neuronal damage and accelerates AD pathology, further compromising BBB integrity [206].

Given that the BBB is a critical barrier protecting the CNS from peripheral insults, its disruption significantly endangers CNS integrity. There are several mechanisms of BBB disruption induced by peripheral inflammation, the most important of which are (1) changes in TJ proteins; (2) damage to endothelial cells; (3) intrusion of peripheral immune cells; (4) alteration of multiple transport pathways and receptors; and (5) activation of astrocytes and microglia.

### 5.1. Changes in Tight Junctions

Inflammation can disrupt the BBB through various pathways, affecting CNS integrity [205]. The BBB serves as an essential immune shield, restricting leukocyte access and preventing CNS-specific antigens from entering the broader immune system. TJs are crucial for maintaining BBB integrity; thus, any changes in TJs directly compromise the barrier. Changes in TJ proteins now serve as common indicators of BBB disruption [206].

#### 5.1.1. Role of Proinflammatory Cytokines

In conditions like T2D, chronic inflammation elevates proinflammatory cytokines that target BBB endothelial cells, compromising TJ proteins and increasing BBB permeability [207]. Although cytokines are large molecules that do not easily cross the BBB [208], they can enter or affect the brain through leaky regions like the choroid plexus or circumventricular organs. Proinflammatory cytokines, such as IL-1β, IL-6, IL-9, IL-17, IFN-γ, TNF-α, and CCL2, decrease TJ expression or cause their mislocalization [209,210,211,212]. Claudin-5 is a particularly critical TJ protein that regulates BBB permeability, whose downregulation during inflammation leads to barrier dysfunction [213]. For instance, peripheral inflammation induced by lipopolysaccharide (LPS) in aged mice degraded TJ proteins, including claudin-5 [214]. Similarly, IL-1β disrupted the continuous distribution of claudin-5 in brain endothelial cells [75]. Additionally, systemic inflammation from LPS exposure led to occludin degradation [215], and peripheral cytokines reduced ZO-1 expression in mice with tumors [216]. Cytokines can also indirectly affect the brain by activating endothelial cells, which release prostaglandins (PGE2) [217].

Other indirect mechanisms affecting TJs involve factors like matrix metalloproteinases (MMPs), ROS, and NF-κB signaling pathways, which will be detailed subsequently [218,219,220,221,222].

#### 5.1.2. Role of Matrix Metalloproteinases

One of the key mechanisms underlying TJ disruption involves the activity of MMPs [223]. MMPs are enzymes that can degrade various components of the extracellular matrix, including TJ proteins like occludin, claudins, and ZO proteins [224]. The degradation of these proteins leads to the disassembly of TJs and a subsequent increase in BBB permeability, allowing the passage of potentially harmful substances into the brain. While MMP activity is tightly regulated under normal physiological conditions, it can be significantly upregulated in response to pathological conditions associated with AD, such as inflammation, ischemia, and oxidative stress [223].

MMPs play a significant role in the pathogenesis of diabetes. Chronic hyperglycemia increases the expression and activity of MMP-9 due to oxidative stress in vascular endothelial cells [225]. Elevated levels of MMP-2 and MMP-9 have been detected in the urine of individuals with T2D, especially those with kidney damage [226]. Further, MMP-2 levels are also increased in the adipose tissue of obese patients [227]. MMP-9 is particularly implicated in the progression of diabetic complications, notably in the retinopathy [228]. Studies indicate that MMP-9 mediates diabetes-induced retinal neuropathy and vasculopathy [229], and it has been associated with the severity of diabetic retinopathy [230].

#### 5.1.3. Role of Oxidative Stress

Hyperglycemia triggers a cascade of events that disrupt the BBB and promote neuroinflammation. It begins with increased mitochondrial respiration in brain cells like endothelial cells, pericytes, and astrocytes, leading to mitochondrial dysfunction and the excessive production of ROS. ROS interact with FoxO targets, causing cellular stress and weakening the TJs that maintain BBB integrity [231,232]. They also impair the folding TJ proteins in astrocytes, disrupting their communication and contributing to BBB breakdown [233]. This damage can lead to pericyte loss and the degradation of astrocytic end-feet, further perpetuating ROS production and oxidative stress [234]. In addition, ROS activate key signaling pathways like JNK and NF-κB [235], which increase the levels of proinflammatory mediators, promoting leukocyte infiltration into the brain [236].

### 5.2. Damage to Endothelial Cells

Endothelial cells (ECs), as primary components of the BBB, are significantly impacted by peripheral inflammation. LPS directly damages the BBB endothelium by inhibiting P-gp activity and promoting the secretion of MMPs, causing membrane abnormalities, endoplasmic reticulum stress, mitochondrial dysfunction, and, ultimately, apoptosis [237,238]. MAPK signaling further mediates EC apoptosis following LPS exposure [239]. The disruption of ECs and subsequent BBB impairment facilitates neurotoxic substance entry into the CNS, raising disease susceptibility [237,240].

Peripheral inflammation also increases the expression of endothelial adhesion molecules, including vascular cell adhesion molecule-1 (VCAM-1) and intercellular adhesion molecule 1 (ICAM-1), as well as E-selectin, enabling immune cell infiltration into the CNS, which is especially notable during aging and chronic inflammation [241,242,243]. Additionally, IL-1β enhances α5 integrin-dependent EC adhesion, disrupting BBB integrity by altering cell–cell junctions and cell–matrix interactions [223].

The NF-κB signaling pathway is pivotal in mediating endothelial cell dysfunction and damage, especially under inflammation and oxidative stress conditions. In response to stimuli such as LPS, TNF-α, interleukins, and ROS, NF-κB becomes activated in ECs. This activation leads to the transcription of multiple proinflammatory genes, including cytokines, chemokines, adhesion molecules (e.g., ICAM-1, VCAM-1), and MMPs [244,245]. As a result, endothelial cells exhibit increased vascular permeability and reduced TJ integrity, including claudin-5 and occludin. These alterations result in the degradation of these proteins and a subsequent reduction in barrier impermeability [246], enhanced leukocyte adhesion and transmigration, and endothelial apoptosis or senescence. This structural breakdown facilitates the passage of plasma molecules and immune cells into the brain parenchyma, promoting a neuroinflammatory state [247].

### 5.3. Penetration of Peripheral Immune Cells

Increased BBB permeability promotes immune cell recruitment into the CNS, amplifying localized inflammation. For instance, IL-1β stimulates astrocytes to secrete VEGF-A, leading to altered occludins and claudin-5 expression that facilitates lymphocyte entry [248]. TNF-α promotes macrophage extravasation through the NF-κB pathway [249]. This immune cell movement relies on adhesion molecules like VCAM-1 and ICAM-1, which are upregulated during inflammation [250].

Peripheral immune cells can also release ROS, MMPs, and NO, which migrate to the CNS and increase BBB permeability by altering TJ proteins [169]. Consequently, reactive astrocytes then produce proinflammatory factors such as IL-1β, IL-6, TNF-α, and COX2, further increasing BBB permeability to harmful transporters and substances, including TNF-α, lysosomal enzymes, and Aβ [251]. This BBB impairment ultimately introduces neurotoxic substances into the CNS, adversely affecting microglia and neurons and increasing the risk of cognitive diseases [169,252].

### 5.4. Changes in Transport Pathways and Receptors

#### 5.4.1. Role of RAGEs

RAGE contributes to neuroinflammation by facilitating Aβ transport across the BBB and directly activating microglia to release proinflammatory cytokines. The RAGE is expressed on various immune-related and non-immune-related cells, including BBB-endothelium cells [253]. Research has found significantly increased RAGE expression in the brains of AD patients [254,255], specifically in regions like the hippocampus and inferior frontal cortex [254]. Two studies used mouse models to investigate the role of RAGE in transporting Aβ into the brain. Deane et al. (2003) demonstrated that RAGE on brain blood vessels facilitated the entry of Aβ [256]. Zhang et al. (2020) further confirmed this by showing that Aβ was not detectable in the brains of mice lacking RAGE [257]. Alternatively, RAGE activation can initiate ROS generation by activating nicotinamide adenine dinucleotide phosphate (NADPH) oxidase [258], which in turn activates NF-kB [259], and NF-kB activation induces IL-1, TNF-α, and interferon-gamma (IFN-γ) [260].

#### 5.4.2. Low-Density Lipoprotein Receptor-Related Protein 1

LRP1 plays a critical role in the integrity of the BBB, and its dysfunction can contribute to its damage. LRP1 facilitates the clearance of Aβ by binding to it and transporting it across the BBB into systemic circulation for hepatic elimination. However, in pathological conditions, the expression and function of LRP1 may decrease, reducing Aβ clearance and promoting its accumulation in the brain, leading to inflammation and neurodegeneration [166]. Also, LRP1 regulates the stability of TJs in the endothelial cells of the BBB. Its dysfunction can increase BBB permeability, allowing inflammatory molecules and immune cells from the periphery to infiltrate the CNS and exacerbating neurovascular inflammation and neuronal damage [167].

### 5.5. Gut Microbiota-Mediated Inflammation

The gut–brain axis is a bidirectional communication system that links the CNS with the gastrointestinal tract, integrating neuroendocrine, immune, and metabolic signals [261]. A key component of this interaction is the gut microbiota, a complex community of microorganisms that influence brain function through bioactive metabolites, cytokines, and neurotransmitters [262,263]. The gut microbiota generates metabolites, including short-chain fatty acids (SCFAs), which exert protective effects by regulating immune responses and oxidative stress on the BBB. SCFAs, particularly butyrate, help strengthen tight junctions between endothelial cells, enhancing BBB integrity and reducing permeability [264,265].

Increasing evidence indicates that changes in microbiota composition may alter BBB permeability, thereby contributing to neuroinflammation and neurodegeneration [266,267]. In dysbiosis conditions, proinflammatory products such as lipopolysaccharide (LPS) can cross the intestinal barrier, compromising its integrity. Gut dysbiosis has been linked to elevated LPS levels, which activate Toll-like receptor 4 (TLR4) and initiate systemic inflammation that disrupts BBB function [264,265].

Notably, microbial metabolites like SCFAs are key in regulating peripheral inflammation, a major contributor to BBB dysfunction [266,268,269]. Furthermore, SCFAs and other bacterial metabolites have shown protective effects on the BBB by reinforcing TJs and regulating immune activity [270,271].

### 5.6. Exosome-Mediated Inflammation

Exosomes (EXOs) are nanosized extracellular vesicles (30–150 nm) secreted by both prokaryotic and eukaryotic cells by fusing multivesicular bodies with the plasma membrane. Unlike broader extracellular vesicles (EVs), EXOs have distinctive roles in intercellular communication by transporting proteins, nucleic acids, and metabolites, significantly influencing physiological and pathological processes. Thanks to their lipid bilayer and small size, they can cross the BBB [272], making them ideal candidates for drug delivery and diagnostic biomarker development in neurological diseases [273].

Their biocompatibility, ability to carry therapeutic loads, and immune-evasive properties make EXOs promising nanocarriers for precision medicine. Current research focuses on engineering EXOs to enhance therapeutic payloads and targeting specificity [274,275]. Moreover, their content, including proteins (e.g., CD9, CD63), lipids, RNA, and DNA, varies depending on the cell of origin and pathological state. Mass spectrometry and specialized databases like ExoCarta [276], Evpedia [277], and Vesiclepedia [278] help in profiling EXO composition for diagnostic and research purposes. EXOs are now recognized as central players in overcoming brain drug delivery barriers, offering a transformative approach to treating neurological disorders [275].

The synthesis and release of EXOs occur through the endosomal sorting complex necessary for transport (ESCRT)-dependent and -independent pathways; the biogenesis and release of EXOs in the CNS begin with endocytosis, where the plasma membrane invaginates inward, incorporating bioactive substances to form early endosomes. This process is facilitated by transferrin receptors, which are later recycled to the membrane. Smaller vesicles bud inward from the endosomal membrane, transitioning early endosomes into late endosomes. Exosomes are released when multivesicular bodies (MVBs) either fuse with lysosomes for degradation or the plasma membrane for secretion. EXO formation is driven by both ESCRT-dependent and ESCRT-independent pathways involving various proteins. Once secreted, EXOs play a vital role in intercellular communication by delivering functional proteins, metabolites, and nucleic acids to target cells [279].

This process is driven by the fusion of MVBs with the plasma membrane, which is orchestrated by proteins such as v-SNAREs (e.g., VAMP7, VAMP8), t-SNAREs (e.g., syntaxin-3, syntaxin-4, SNAP23), and Rab GTPases (e.g., Rab27a/b, Rab35). These components ensure vesicle targeting, docking, and membrane fusion. EXO release can be calcium-dependent in some hematopoietic cells, like T and mast cells [275,279].

EXOs play a central role in neurodegenerative diseases such as AD, Parkinson’s disease, and amyotrophic lateral sclerosis. In AD, EXOs contribute to pathology by facilitating the transport of Aβ plaques from the extracellular space into the intracellular environment. They also serve as vehicles for the intercellular transfer of Aβ, enabling its propagation from one cell to another. Conversely, they also have neuroprotective functions by facilitating the clearance of accumulated toxins, such as Aβ and α-synuclein, thereby alleviating the cellular burden [280]. EXOs are also involved in neuroinflammation, where they modulate glial activation. Novel therapeutic strategies, such as IL-1β-stimulated EXOs embedded in injectable hydrogels, have shown promise in reducing neuroinflammation and promoting neuronal recovery [281].

Recent advances in AD therapy have increasingly focused on nanomedicine, especially EXO-based strategies, due to their natural ability to transport bioactive molecules and cross the BBB [282]. In AD, characterized by Aβ plaque accumulation and tau tangles, EXOs play a dual role: they contribute to disease progression by spreading pathological protein and offer neuroprotective potential by removing toxic aggregates and delivering protective agents [283,284].

Multiple preclinical studies have explored engineered EXOs for therapeutic applications in AD. It has been demonstrated that 3D graphene scaffolds used to culture hUMSCs produced EXOs rich in beneficial proteins and miRNAs. These 3D EXOs decreased β-secretase, increased α-secretase expression, and reduced Aβ levels, inflammation, oxidative stress, and microglial activation in vitro and in vivo [285].

Another study showed that mesenchymal stem-cell-derived EXOs restored synaptic gene expression, reduced Aβ, improved brain glucose metabolism, and enhanced memory in AD models. Brain cell phase balance was also regulated after treatment [286]. A modified EXO using rabies viral glycoprotein (RVG) peptide enhanced cognitive outcomes, reduced plaque burden, and shifted cytokine profiles toward anti-inflammatory states in APP/PS1 mice [287]. A research study designed quercetin-loaded EXOs, which effectively inhibit tau hyperphosphorylation and improve memory in okadaic acid-induced AD rats [288].

## 6. Glial Activation and Neuroinflammation Induced by Peripheral Inflammation

BBB impairment induced by chronic peripheral inflammation and gut microbiota dysbiosis activates microglia, promoting the accumulation of neurotoxic proteins [289,290] and reducing Aβ clearance mechanisms, which promotes accumulation in the hippocampus [291,292]. Similarly, it exacerbates tau pathology, contributing to neuronal loss and cognitive decline [293]. On the other hand, recent research shows that gut microbial changes can modify systemic cytokine levels, affecting Aβ deposition and clearance mechanisms in the brain [294,295].

### 6.1. Microglial Activation

Peripheral inflammation has been demonstrated to induce neurodegeneration, a process associated with the chronic activation of microglia. For instance, inflammatory cytokines, such as TNF-α, IL-6, and IL-1β, have promoted microglial activation and changed them to a proinflammatory M1 phenotype [296,297]. This leads to the increased production of ROS and NO, which in turn causes oxidative stress, synaptic dysfunction, and neuronal apoptosis [298]. Likewise, BBB dysfunction allows peripheral immune cells and inflammatory mediators to enter the CNS, exacerbating neuroinflammation and neuronal injury [255].

Microglia also influence BBB disruption through inflammation-mediated activation [299]. M1 microglia disrupt BBB integrity primarily through the production of inflammatory mediators, promotion of immune cell trafficking, and oxidative stress, mediated by receptors such as TLR-4, IFN-γ receptor complex, GM-CSF receptor, and enzymes like COX2 [123,124,125,126,127,128]. Proinflammatory cytokines (e.g., TNF-α, IL-1β, IL-6, IL-12, CCL2, and CXCL10) released by M1 microglia alter TJ proteins (claudin-5, occludin, ZO-1, and ZO-2) and key transporters like P-gp at the BBB, promoting immune cell infiltration, as observed in stroke [28,30,101,129,130,131,132,133,134]. M1 activation also increases ROS production, oxidative stress, and iNOS expression during peripheral inflammation and stroke [300,301]

### 6.2. Astrocyte Activation

Astrocytes are essential for maintaining BBB integrity and regulating its permeability. During BBB dysfunction, astrocytes undergo significant changes, becoming “reactive astrocytes”. Their reactive state is characterized by enlarged cell structures; the overexpression of proteins like GFAP, nestin, and vimentin; and increased production of inflammatory cytokines [302] that promote an inflammatory response and can disrupt neuronal function [303]. Reactive astrocytes secrete mediators such as VEGF-A, which activates endothelial nitric oxide synthase (eNOS) signaling in endothelial cells (ECs), reducing the expression of occludin and claudin-5 and facilitating immune cell entry into the CNS [114,115], which damages the BBB’s endothelial lining [304]. This damage activates inflammatory pathways (NLRP3 inflammasome and NF-κB) that increase proinflammatory molecules and activate matrix metalloproteinase 9 (MMP9) [305]. Moreover, LPS induced by gut microbiota triggers astrocyte proliferation and activation, structural changes in end-feet, and altered gene expression, indirectly compromising BBB integrity [118,119]. Finally, this leads to structural changes in the BBB, reduced TJ protein expression, and overall BBB dysfunction [306,307].

### 6.3. Neuroinflammation

Neuroinflammation is currently recognized as a crucial player in synapse damage and brain dysfunction. The central inflammatory response contributes to cognitive dysfunction and neurodegeneration in AD [30,34,308,309]. At the onset of AD, microglia are essential for Aβ clearance through phagocytosis, enzymatic degradation, and interaction with the BBB via microglia scavenger receptors [310], preventing the formation of amyloid plaques in the brain [311]. Microglia internalize and degrade Aβ through phagocytosis and receptor-mediated endocytosis. Receptors such as scavenger receptors (e.g., SR-A, CD36), Toll-like receptors (TLRs) (e.g., TLR2, TLR4), and LRP1 facilitate this process by recognizing and binding to Aβ, promoting its uptake [25]. Once internalized, microglia utilize lysosomal enzymes to break down Aβ into non-toxic fragments. These enzymes include neprilysin (NEP), IDE, MMPs, and cathepsins [312]. Finally, microglia contribute to Aβ transport out of the brain by interacting with the BBB to facilitate its clearance via LRP1 [25].

Nevertheless, Aβ oligomers can induce neuroinflammation in pathological conditions. Microglia activation by Aβ oligomers causes a release of TNF-α. It stimulates TNF-α receptor 1 (TNFR-1), which induces JNK activation stress kinase, which subsequently blocks the intracellular actions of insulin by pSer-IRS-1. Akt subsequently reduces GSK-3β phosphorylation [313,314], leading to an upregulation in tau phosphorylation and Aβ formation, which impacts synaptic dysfunction, synaptic plasticity, and loss of synapses. Prolonged activation releases proinflammatory cytokines, which initiate a proinflammatory cascade and contribute to neuronal damage and loss [315,316]. At the same time, Aβ-stimulated persistent microglial activation via the CD36 receptor [317], TLR4, [318], and RAGEs [319] can increase Aβ production and decrease Aβ clearance, causing neuronal damage. When Aβ-induced microglial activation is inhibited, the production of inflammatory cytokines is decreased, as well as Aβ storage [320] and memory improvement [321,322].

## 7. Brain Insulin Resistance and the Impact on Neurodegeneration

### 7.1. Brain Insulin Function

Chronic neuroinflammation becomes detrimental, causing progressive tissue damage and disrupting insulin signaling pathways, and leading to brain insulin resistance. Brain insulin resistance contributes to Aβ formation and tau phosphorylation. Insulin is found at considerably lower cerebrospinal fluid (CSF) concentrations than plasma [323]. Despite this, correlations between CSF and plasma insulin levels indicate that most brain insulin originates from circulating pancreatic insulin [324]. Insulin crosses the BBB via two main mechanisms: active transport through insulin-specific transporters in endothelial cells and passive diffusion in circumventricular regions [325]. Unlike in peripheral tissues, insulin in the brain does not regulate glucose uptake but instead plays critical roles in age-related neurodegeneration, brain plasticity, and cognitive processes such as memory, learning, and executive function [32,326,327,328]. It also regulates apoptosis, lipid metabolism, cerebral blood flow, glial inflammatory responses, oxidative stress, Aβ clearance, tau phosphorylation, and neurotransmission by modulating glutamatergic and GABAergic receptor trafficking, which are essential for synaptic plasticity and memory [329,330].

### 7.2. Brain Insulin Signaling

Insulin binds to IR inside the brain, forming an insulin–receptor complex that undergoes transcytosis to the brain’s endothelial cells [331]. Neuronal IR is predominantly located in the olfactory bulb, hippocampus, neocortex, hypothalamus, and cerebellum [332]. The signaling mechanism resembles that of peripheral IRs. The receptor’s intrinsic tyrosine kinase activity phosphorylates intramembrane domains upon insulin binding, creating docking sites for IRS and Shc proteins. IRS proteins, which are rich in Ser/Thr/Tyr residues, undergo phosphorylation, which regulates their function [29]. Insulin binding to neuronal IRs triggers autophosphorylation of Tyr residues, activating by phosphorylation of IRS-1 on specific Tyr residues (Tyr1158/1162/1163). This activation initiates signaling pathways, including the PI3K-Akt pathway [333]. PI3K converts PIP2 to PIP3, recruiting Akt to the plasma membrane, where it is phosphorylated by PDK1 and PI3K, which activates its Thr residues [29]. The PI3K/Akt pathway supports axonal growth, regeneration, and protein synthesis via mTOR activation at Ser2448 [334].

Additionally, phosphorylated Akt modulates various effectors, including AS160, which activates Thr642 to facilitate the translocation of GLUT4 vesicles to the plasma membrane for glucose uptake. Hexokinase-II (HKII), activated by Akt, plays a critical role in glucose metabolism and mitochondrial function. On the contrary, Akt inhibits FoxO transcription factors by phosphorylation, which regulates genes involved in processes ranging from cell survival to apoptosis [335].

**Figure 5:**
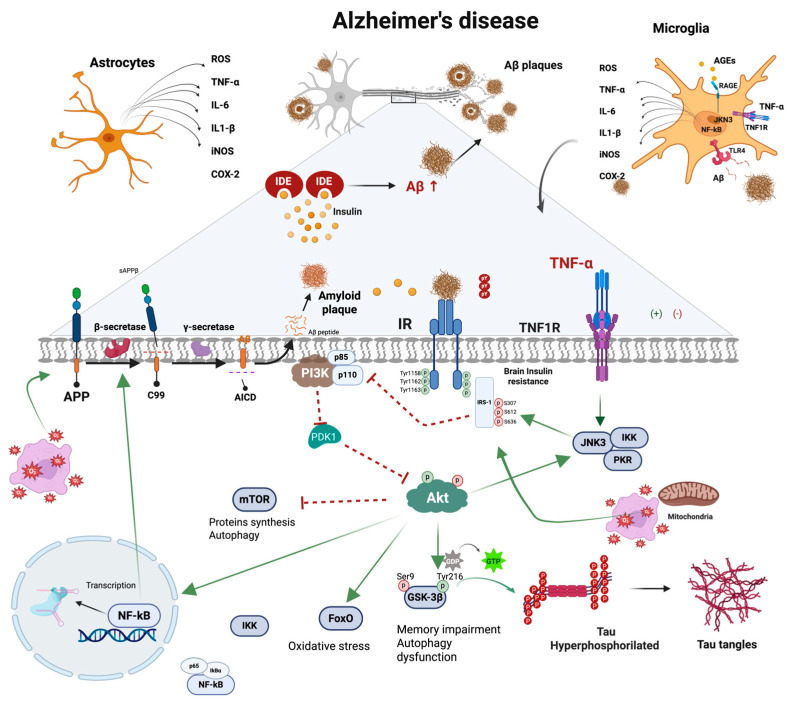
Mechanisms of insulin resistance, neuroinflammation, and tau pathology in Alzheimer’s disease. The interaction between insulin resistance, Aβ accumulation, neuroinflammation, and tau hyperphosphorylation plays a central role in AD. Astrocytes and microglia drive neuroinflammation by releasing proinflammatory cytokines such as TNF-α, IL-6, and IL-1β, along with ROS and iNOS. The activation of microglial RAGE by Aβ AGEs triggers signaling cascades involving TLR4, JNK3, and NF-κB, amplifying inflammatory responses and oxidative stress. Insulin signaling is severely impaired in AD, leading to brain insulin resistance. IDE, which plays a key role in the clearance of insulin and Aβ, becomes dysfunctional, contributing to Aβ accumulation and amyloid plaque formation. The disruption of IR signaling results in diminished phosphorylation of key downstream effectors, including PI3K, PDK1, and Akt, exacerbating oxidative stress, memory impairment, and autophagy dysfunction. TNF-α signaling through its receptor TNFR1 activates JNK3, IKK, and PKR, further inhibiting Akt and enhancing GSK-3β activity—a critical driver of tau hyperphosphorylation and neurofibrillary tangle formation. Additionally, dysregulation of mTOR, NF-κB, and FoxO pathways worsens oxidative stress, disrupts protein synthesis, and impairs autophagy, all contributing to progressive neurodegeneration in AD. Solid Arrows: indicates activation or conversion step in the signaling pathway. Dotted lines: indicates inhibitory effect. AD: Alzheimer’s disease; Aβ: amyloid beta; TNF-α: tumor necrosis factor alpha; TNFR1: TNF-receptor 1; IL-1 β: interleukin-1β; IL-6: interleukin-6; ROS: reactive oxygen species; iNOS: inducible nitric oxide synthase; RAGE: receptor for advanced glycation end products; AGEs: advanced glycation end products; APP: amyloid precursor protein; IDE: insulin-degrading enzyme; PI3K: phosphoinositide 3-kinase; PDK1: phosphoinositide-dependent kinase-1; Akt: protein kinase B, PKB; JNK3: c-Jun N-terminal kinase 3; IKK: IκB kinase complex; PKR: protein kinase R; GSK-3β: glycogen synthase kinase-3 beta; mTOR: mammalian target of rapamycin; NF-κB: nuclear factor kappa-light-chain-enhancer of activated B cells; FoxO: forkhead box O transcription factor.

Akt likewise inactivates GSK-3β, which is expressed in the CNS and mainly expressed in axons, playing a crucial role in phosphorylating the tau protein [336]. The activity of GSK-3β can be regulated by phosphorylation at serine 9 (pSer 9; inactivation) and tyrosine 216 (pTyr 216 activation) [324]. In AD, the PI3K/Akt pathway, which regulates GSK-3β inactivation, is altered, leading to increased GSK-3β activity [337]. Elevated levels of pTyr216 GSK-3β or its overactivity increase tau phosphorylation, leading to microtubule disassembly. Its processes cause disturbances in axonal transport, hippocampal neurodegeneration [338], and learning impairment [339]. GSK-3β can phosphorylate tau at 42 sites [340], and its activity is correlated with the accumulation of NFTs in AD brains [341] (Figure 5).

Furthermore, GSK-3β modulates intrinsic cellular apoptosis pathways, which the Aβ peptide can influence. GSK-3β could inhibit Aβ production through the downregulation of BACE1 mRNA, which inhibits BACE1 activity and amyloid precursor protein (APP), thereby reducing Aβ peptide production [342]. In a double transgenic AD model in mice, it was demonstrated that the inhibition of GSK3β signaling regulates BACE1 expression and that this is dependent on NF-κB; as a result, there was reduced Aβ and neuritic plaque formation, as well as reversed memory deficits. GSK3β also plays a role in APP processing by modulating γ-secretase activity, facilitating Aβ production. BACE1 cleavage results in reduced APP and Aβ production by decreasing BACE1 gene transcription and expression [342] (Figure 5).

In this context, insulin acts as a neuroprotective agent in two ways. First, it competes against Aβ oligomers for binding to a common binding site on the neuronal surface [32]. Second, it reduces Aβ aggregation by decreasing the phosphorylation of its APP; this increases the IDE, which is the enzyme involved in the clearance of both insulin and Aβ [343], and reduces the formation of its oligomers [335]. Finally, other targets that could also be affected are the downstream effectors of impaired Akt, FoxO, NF-kB, JNK1, and mTORC1 activation, which could cause oxidative stress, neuroinflammation, mitochondrial dysfunction, and autophagy [33,49,344,345] (Figure 5).

### 7.3. Brain Signaling Disruption

Several factors, including an HFD, astrocyte activation [346], obesity, inflammation, diabetes, and elevated triglyceride levels, can disturb the insulin transport process and signaling receptor [334]. IRS-1 can be phosphorylated on Ser residues, promoting uncoupling between IR and IRS-1. Like peripheral insulin resistance, this molecular effect is also the basis of the central insulin resistance phenomenon. Therefore, brain insulin resistance comes from the downregulation of IR or an incorrect activation of the insulin signaling cascade, mainly driven by IRS-1 inhibition [324]. Indeed, IR downregulation decreases the pool of IR in the plasma membrane available to bind insulin. Instead, the inhibition of IRS-1, which is mediated by the phosphorylation of specific serine residues (e.g., pSer307, pSer312, and pSer636), leads to the uncoupling of IRS-1 from the IR, finally responsible for the inability of insulin to promote the PI3K/Akt signaling pathway [324]. Thus, inactive Akt that increases GSK-3β activation can lead to tau phosphorylation.

Additionally, it is implicated in the increased deposition of Aβ and inflammation [32]. Studies have revealed that GSK3β is linked to memory loss and learning impairment. Its activation and overexpression lead to a decrease in long-term potentiation (LTP) and an increase in long-term depression (LTD) by modulating N-methyl-D-aspartate receptor (NMDA) receptors [347,348]. Lithium-induced inhibition of GSK3β has shown protective effects against neurodegeneration, significantly reducing phospho-tau levels, spatial learning deficits, and memory impairments in a rat model [188]. Consequently, neuronal insulin resistance could affect AD-related pathology and symptoms [330,349]. Insulin insensitivity or neuronal insulin resistance occurs in the hippocampus and cortex [350,351]. The inactivation of HKII could trigger mitochondrial failure, leading to increased oxidative stress and favoring APP amyloidogenic cleavage and eventually, Aβ buildup [324].

The above is supported by evidence that suggests that brain insulin signaling is analogous to peripheral insulin resistance and its elemental role in the pathogenesis of AD. Various researchers have associated the reduction in IRS-1 levels and the rise in IRS-1pSer levels, indicating impairment in the insulin signaling pathway [28]. They found diminished insulin signaling in the IR/IRS-1/PI3K pathway and reduced responses to insulin-like growth factor 1(IGF-1) in the IGF-1/IRS-1,2-PI3K pathway. Elevated levels of phosphorylated IRS-1 at specific serine residues (IRS-1pS616 and IRS-1 pS636/639) were identified as potential biomarkers of this brain insulin resistance in the hippocampal formation in AD. These markers increased progressively from normal to MCI in AD cases, regardless of diabetes or APOE ε4 status. The levels of these markers were positively correlated with oligomeric Aβ plaques and negatively associated with cognitive functions like episodic and working memory. They suggested that brain insulin resistance is an early and common feature in AD, likely influenced by Aβ oligomers, and contributes to cognitive decline independently of other AD pathologies [28].

At the same time, another study reported that elevated levels of IRS-1pSer and activated JNK were found in the brain tissues of AD patients, like peripheral tissue changes in diabetes patients. Likewise, Aβ oligomers deposited in the AD brains were shown to activate the JNK/TNF-α pathway and inhibit physiological IRS-1 tyrosine phosphorylation in cultured hippocampal neurons [319]. The same authors also demonstrated these effects in transgenic mice, a model of AD. Additionally, the intracerebroventricular injection of Aβ oligomers in cynomolgus monkeys activated hippocampal pSer-IRS-1 and JNK [319]. Elevated levels of IRS-1pSer612/636, which play a crucial role in insulin resistance, have been observed in the brains of AD transgenic mice (APP/PS1 mice, [352]).

In neural-derived blood exosomes obtained from preclinical subjects (individuals with asymptomatic amyloidosis who are cognitively intact 1–10 years before their AD diagnosis) or AD patients, the total level of IRS-1 is decreased to a lesser extent compared to the level of IRS-1pTyr. Conversely, the IRS-1pSer312 level appears to be increased. The IR index, which represents the ratio of IRS-1pSer312 to IRS-1pTyr, is higher in preclinical subjects or AD patients than in age- and gender-matched controls [353]. A study found that AD brain atrophy is linked to IRS-1 expression, showing a positive correlation with IRS-1pTyr and a negative correlation with IRS-1pSer312 in a spatial pattern [354]. Notably, the connection between brain insulin signaling and AD pathogenesis has shown that reduced levels of IRS-1 expression are linked to phosphorylated tau proteins. Moreover, abnormal IRS-1 hyperphosphorylation is associated with tau hyperphosphorylation [354].

An additional study of postmortem tissue across different stages of AD revealed early disruptions in mTOR signaling and autophagy, indicated by increased Aβ1–42 levels and decreased autophagy markers. Hyperactivation of the PI3K/Akt/mTOR pathway was observed in cases of MCI and AD but not in preclinical AD (PCAD). This hyperactivation was linked with raised insulin resistance biomarkers and GSK3β, a kinase involved in tau phosphorylation [355]. In transgenic mouse models of AD, the participation of the insulin signaling pathway has also been studied. A study in a mouse model of AD shows a reduction in the activity of biliverdin reductase-A, an enzyme that participates in cell growth, which was triggered by oxidative stress, which precedes the accumulation of Aβ, tau pathology, and the elevation of TNF-α. This impairment leads to the prolonged activation of IRS-1, which triggers harmful feedback mechanisms to mitigate excessive IRS-1 overactivity and brain insulin resistance [356]. In additional research with 3xTg-AD and Tg2576 mice, an alteration of the brain insulin signaling pathway has been observed, with modifications in the levels of IRS-1, p-PI3K, p-Akt, and GSK3β [357].

Altogether, evidence connecting peripheral inflammation to BBB dysfunction highlights the importance of targeting peripheral inflammation to develop effective neuroprotective therapies that preserve and restore BBB function.

## 8. Emerging Therapeutic Strategies

Current research emphasizes the significant impact of metabolic dysfunctions—particularly insulin resistance and systemic inflammation—and the dysbiosis of the gut–brain axis on BBB integrity. Hence, enhancing the integrity of a compromised BBB involves targeting various cellular and molecular components of the endothelial cell. Pharmacological strategies generally aim to counteract the specific pathological changes that lead to barrier dysfunction [358].

Key mechanistic approaches emerging from recent research include reinforcing TJ structure and mitigating local inflammation and oxidative stress by targeting key inflammatory and regulatory pathways involved in its dysfunction. One prominent approach involves cytokine inhibitors, which target specific proinflammatory cytokines that drive inflammation and increase BBB permeability. Among these, TNF-α inhibitors, such as etanercept and adalimumab, which are commonly used in autoimmune diseases, are currently under evaluation in clinical trials for conditions like multiple sclerosis (MS) and AD due to their potential to reduce BBB leakage and protect neuronal tissue [359].

Closely related are NF-κB inhibitors, which aim to block the activity of the NF-κB pathway—a central regulator of inflammatory cytokine expression and TJ destabilization in the BBB. Natural compounds such as curcumin have shown promise in inhibiting NF-κB signaling, offering anti-inflammatory benefits [360,361]. Moreover, the upregulation of Cyclophilin A (CypA) in pericytes activates the NF-κB–MMP9 cascade, leading to TJ degradation. The pharmacological inhibition of CypA using agents such as cyclosporine A or alisporivir has been shown to block this pathway and restore BBB integrity in experimental models [362].

Another promising avenue involves inhibitors of coagulation factors, particularly Factor XI (FXI), which has been implicated in neuroinflammatory diseases such as MS. During inflammation, FXI may leak into the CNS, exacerbating BBB permeability and inflammation. Preclinical studies using anti-FXI monoclonal antibodies in experimental autoimmune encephalomyelitis (EAE) models have demonstrated reduced CNS inflammation and BBB disruption, suggesting that targeting inflammation-associated coagulation factors may help preserve BBB function [363].

In addition to anti-inflammatory strategies, therapies aimed at modulating the function of BBB transporters and receptors offer another level of intervention. This includes restoring the activity of proteins such as LRP1, critical for Aβ clearance and BBB stability [364,365]. Similarly, inhibiting the receptor for RAGE, which contributes to inflammation when activated by Aβ and other ligands, has therapeutic potential [366].

Parallel to these molecular approaches, nanoparticle and exosome-based therapies—particularly those utilizing exosomes derived from mesenchymal stem cells (MSCs) or neural progenitor cells—are gaining attention for their ability to cross the BBB and deliver therapeutic molecules directly into the CNS. These exosomes can carry anti-inflammatory miRNAs and proteins, modulate microglial polarization from a proinflammatory (M1) to an anti-inflammatory (M2) state, suppress pathways such as NF-κB, and potentially restore the expression of tight junction proteins, thereby contributing to BBB repair [367,368,369].

An emerging area of interest is modulating the gut–brain axis, as increasing evidence suggests that gut dysbiosis and systemic inflammation play a significant role in BBB disruption. Microbiome-targeted interventions—such as the administration of probiotics and prebiotics—can attenuate peripheral immune activation and metabolic dysfunction, ultimately reducing neuroinflammation and supporting the maintenance of BBB integrity [370,371,372,373].

Together, these therapeutic strategies reflect the multifaceted nature of BBB dysfunction in neurodegenerative diseases and emphasize the importance of targeting systemic and CNS-specific pathways to preserve brain homeostasis.

### 8.1. Therapeutic Approaches in Metabolic Syndrome

Given the strong association between metabolic syndrome and AD, emerging clinical strategies aim to mitigate AD risk by targeting metabolic dysfunctions. Therapeutic interventions such as metformin, an insulin-sensitizing agent, have demonstrated cognitive benefits and reduced AD-related pathology through mechanisms involving improved insulin signaling and reduced neuroinflammation [374,375]. GLP-1 receptor agonists, including *liraglutide* and *semaglutide*, are under investigation for their neuroprotective effects. They show promise in enhancing brain insulin signaling and attenuating inflammation [376].

Moreover, addressing peripheral and central inflammation with anti-inflammatory agents, such as TNF-α inhibitors and selective nonsteroidal anti-inflammatory drugs (*NSAIDs*), may reduce neurodegenerative progression in at-risk populations [313]. Lifestyle interventions—such as adherence to the Mediterranean diet, regular physical activity, and weight management—have also been associated with improved metabolic profiles and cognitive outcomes [377]. Eventually, novel strategies targeting the BBB, including the modulation of LRP1 and RAGE transporters, seek to restore barrier integrity and reduce Aβ accumulation [378].

### 8.2. Discrepancies in BBB Permeability Between AD Animal Model and AD Human Brain

BBB dysfunction is known to contribute to AD. Therefore, research is increasingly focused on uncovering the mechanisms responsible for BBB permeability alterations in AD, exploring its role in disease progression, and evaluating the BBB as a promising target for therapeutic strategies [377]. However, significant differences in BBB permeability have been identified between animal models of AD and humans [379,380].

Differences between the rodent and human BBB have been reported in TJs, specifically claudin-5, occludin, and ZO-1, which have been reported to have higher mRNA expression and transporter expression and function in mouse brain endothelial cells compared to human brain endothelial cells [381]. In addition, a comparison of protein-level expression of transporters in brain microvessels identified a higher expression of some transporters, including ABC (P-gp) and SCL transporters, in rats compared to humans [382].

Real-time brain imaging also revealed differences in P-gp-dependent drug uptake between rat and human brains, with brain concentrations of P-gp substrates found to be higher in humans than in rats [383]. These data demonstrate higher BBB permeability in humans than in rodents, suggesting that drug delivery experiments cannot be directly translated from rodents to humans. Interestingly, differences between humans and other primates are more insignificant, so non-human primates potentially provide a more accurate model for human drug delivery than rodents [382].

Postmortem human studies and cellular model studies have also identified reduced integrity of brain endothelial cells, altered expression of TJ and AJ proteins in brain endothelial cells, and reduced LRP1 expression in the brain endothelium of AD patients [384]. LRP1, the primary receptor that facilitates Aβ clearance from the brain, and its reduced expression suggest a possible mechanism for Aβ accumulation in AD [384]. Conversely, APPSw/0 mice and other APP models have reported low levels of LRP1 [385]. In various murine models of AD, such as PS2-APP, tau transgenic, and APOE4 knocking mice, the BBB remains intact without significant disruption, sufficient to allow the passage of antibodies or molecules of various sizes [386].

These findings contradict other studies showing BBB degradation in various animal models of AD, including capillary leakage, degeneration, and loss of brain capillary pericytes, endothelial cells, vascular smooth muscle cells (VSMCs), and TJ proteins, as well as altered expression of transporters and receptors [381,387,388,389]. The observed discrepancies may be due to variations in the techniques used to assess BBB permeability, the specific animal models employed, and the stage of disease progression examined [390].

## 9. Conclusions

This review highlights that, beyond the classical amyloid and tau hypotheses, metabolic disturbances particularly accelerate neuronal dysfunction, synaptic loss, and cognitive decline. The complex association between peripheral insulin resistance, chronic inflammation, and neurodegeneration has been proposed as a potential contributor to the development of AD. The BBB protects the brain and maintains its internal balance, functioning as a selective filter between the blood and the CNS. However, when its permeability changes, it becomes a key factor in the development and progression of neurodegenerative diseases such as AD.

**Figure 6:**
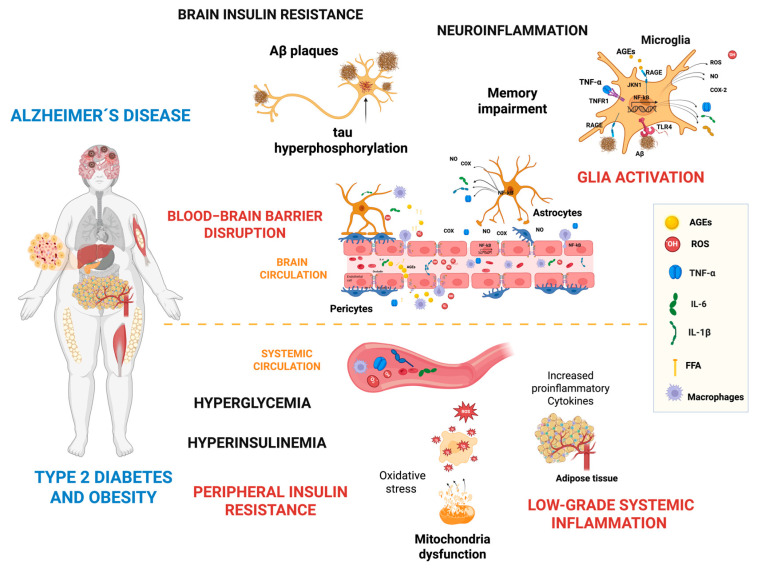
Interaction between type 2 diabetes, obesity, metabolic syndrome, and the development of Alzheimer’s disease through mechanisms involving insulin resistance, neuroinflammation, and blood–brain barrier dysfunction.

Chronic conditions such as diabetes, obesity, and metabolic syndrome, which are characterized by inducing insulin resistance and chronic peripheral inflammation, mainly by activating immune cells and overproducing proinflammatory cytokines, could compromise the integrity of the BBB. The breakdown of this selective barrier permits peripheral inflammatory mediators to infiltrate the CNS, activating astrocytes and microglia, which releases additional neurotoxic factors, NO, ROS, and cytokines through NF-κB signaling and perpetuates a self-sustaining neuroinflammatory loop. Ultimately, these mechanisms contribute to brain insulin resistance and neuroinflammation to promote the pathological hallmarks of AD, including Aβ accumulation and tau hyperphosphorylation (Figure 6).

Understanding these related processes underlines the need for integrative therapeutic strategies targeting systemic inflammation, BBB protection, and gut–brain axis modulation to prevent or delay the onset of neurodegeneration in vulnerable populations. Exploring anti-inflammatory approaches—including cytokine and NF-kB inhibitor agents, RNA interference, gene therapy, nanoparticles, and gut microbiota modulation—has demonstrated promising potential in restoring BBB integrity and protecting neurons from damage.

Nevertheless, searching for new treatments requires a multidisciplinary strategy to fully address these intricate issues and develop more specific and successful neuroprotective therapies customized for each patient’s unique requirements.

## Figures and Tables

**Table 1:** Primary endothelial transportation and regulation mechanisms.

Mechanism	Description and Function	Transporter and Key Transported Molecules
ABC Transporters	Active transport using ATP to efflux molecules from the brain.	- P-gp: Effluxes Aβ, drugs, and xenobiotics. - ABCA1: Transports cholesterol and phospholipids to ApoE, influencing lipid content in the brain.
SLC Transporters	Facilitate diffusion of essential molecules into the brain.	- GLUT1: Transports glucose across the BBB. - ASCT1: Transports alanine, serine, cysteine, and threonine. - SLC39: A zinc transporter; removes toxic substances like Aβ.
MFSD2A	LPC symporter that regulates brain lipids.	- DHA: An essential omega-3 fatty acid for neuronal function.

ABC: ATP-binding cassette; P-gp: P-glycoprotein; Aβ: amyloid beta; SLC: solute carrier; BBB: blood–brain barrier; LPC: sodium-dependent lysophosphatidylcholine; DHA: docosahexaenoic acid; MFSD2a: major facilitator superfamily domain-containing protein 2a.

**Table 2:** Receptor-mediated transport and regulation.

Receptor	Description and Function	Ligands
LRP1	Scavenger receptor on endothelial cells and pericytes that mediates Aβ clearance from the brain to peripheral circulation.	LDL, Aβ
RAGE	Transports Aβ and neuropeptides across the BBB into the brain; expression is regulated by ligand concentration.	AGEs, Aβ, S100B, Mac-1, HMGB1, amphoterin

LRP1: low-density lipoprotein receptor-related protein 1; Aβ: amyloid beta; LDL: low-density lipoprotein; RAGE: receptor for advanced glycation end products; BBB: blood–brain barrier; AGEs: advanced glycation end products; S100B: S100 calcium-binding protein B; Mac-: macrophage-1 antigen; HMGB1: high-mobility group box 1.

## Data Availability

Not applicable.

## Abbreviations

The following abbreviations are used in this manuscript:

ABC	ATP-binding cassette
ACh	acetylcholine
AChT	acetylcholine transferase
AD	Alzheimer’s disease
AGEs	advanced glycation end products
Akt	protein kinase B
APP	amyloid precursor protein or amyloid-β precursor protein
Aβ	amyloid beta or beta amyloid
BACE-1	β-secretase
BMVECs	brain microvascular endothelial cells
CCR2	C-C chemokine receptor type 2
CD36	cluster of differentiation 36
CNS	central nervous system
COX	cyclooxygenase
CRP	C-reactive protein
CSF	cerebrospinal fluid
DAG	diacylglycerol
DHA	docosahexaenoic acid
FFAs	free fatty acids
FoxO	forkhead box-containing transcription factor
GLUT4	glucose transporter type 4
GSK-3β	glycogen synthase kinase-3β
HFD	high-fat diet
HMGB1	high-mobility group box 1
IAPP	amyloid polypeptide
ICAM-1	intercellular adhesion molecule 1
IDE	insulin-degrading enzyme
IFN-γ	interferon-gamma
IGF-1	insulin-like growth factor 1
IKK	IκB kinase
IL-10	interleukin-10
IL-1β	interleukin-1β
IL-6	interleukin-6
iNOS	inducible nitric oxide synthase
Ins2 gene	insulin gene
IR	insulin receptor
IRS-1	insulin receptor substrate 1
IRS-1pSer/Thr	serine/threonine phosphorylation of IRS-1
IRS-1pTyr	phosphorylation of the tyrosine residue of IRS-1
IRS1-4	insulin receptor substrates 1-4
IκB	inhibitor kappa B
JNK	c-Jun N-terminal kinase
LDL	low-density lipoprotein
LPC	sodium-dependent lysophosphatidylcholine
LRP1	low-density lipoprotein receptor-related protein 1
LTD	long-term depression
LTP	long-term potentiation
Mac-1	macrophage-1 antigen
MAPK	mitogen-activated protein kinase
MCI	mild cognitive impairment
MCP-1	monocyte chemoattractant protein
MFSD2a	major facilitator superfamily domain-containing protein 2a
MMP9	matrix metalloproteinase 9
MMPs	matrix metalloproteinases
mTORC1	mammalian target of rapamycin
NADPH	nicotinamide adenine dinucleotide phosphate
NEP	neprilysin
NF-kB	nuclear factor kappa B
NFTs	intracellular neurofibrillary tangles
NMDA	N-methyl-D-aspartate receptor
NO	nitric oxide
NOS	nitric oxide synthase
P-gp	P-glycoprotein
PCAD	preclinical AD
PDK1	phosphoinositide-dependent kinase 1
PGE2	prostaglandins
PH	pleckstrin homology
PI3K	phosphatidylinositol 3-kinase
PIP2	phosphatidylinositol (4,5)-biphosphate
PIP3	phosphatidylinositol (3,4,5)-trisphosphate
PKC	protein kinase C
PRAS40	proline Rich Akt Substrate, 40 kDa
PTB	phosphotyrosine-binding domain
RAGE	receptor for advanced glycation end products
RHD	Rel homology domain
RNS	nitrogen species
ROS	reactive oxygen species
S6K	protein S6 kinases
SLC	solute carrier
SR-A	type A scavenger receptor
SREBP1	sterol regulatory element binding protein
T2D	type 2 diabetes
TJs	tight junctions
TLRs	Toll-like receptors
TNF-α	tumor necrosis factor-alpha
TNFR-1	tumor necrosis factor-alpha receptor 1
VCAM-1	vascular cell adhesion molecule-1
VEGF	vascular endothelial growth factor
ZO	zonula occludens
